# ORF1ab codon frequency model predicts host-pathogen relationship in orthocoronavirinae

**DOI:** 10.3389/fbinf.2025.1562668

**Published:** 2025-03-18

**Authors:** Phillip E. Davis, Joseph A. Russell

**Affiliations:** MRIGlobal, Gaithersburg, MD, United States

**Keywords:** machine learning, feature selection, genotype-to-phenotype, viruses, bioinformactics

## Abstract

Predicting phenotypic properties of a virus directly from its sequence data is an attractive goal for viral epidemiology. Here, we focus narrowly on the Orthocoronavirinae clade and demonstrate models that are powerfully predictive for a human-pathogen phenotype with 76.74% average precision and 85.96% average recall on the withheld test set groups, using only Orf1ab codon frequencies. We show alternative examples for other viral coding sequences and feature representations that do not perform well and discuss what distinguishes the models that are performant. These models point to a small subset of features, specifically 5 codons, that are critical to the success of the models. We discuss and contextualize how this observation may fit within a larger model for the role of translation in virus-host agreement.

## Introduction

There are several examples of modeling efforts attempting to assess either the host range or zoonotic potential of RNA viruses broadly, and Coronaviruses specifically in the wake of COVID-19 (Babayan et al., 2018; Young et al., 2020; Brierley and Fowler, 2021; Mollentze et al., 2021; Gonzalez-Isunza et al., 2023). There are many commonalities between these approaches. Naturally, due to its role in receptor binding, Spike protein is often the focus of these efforts when analyzing properties such as nucleotide or amino acid composition. Limitations to focusing on Spike protein exclusively have been described previously (Brierley and Fowler, 2021; Mollentze et al., 2022). Other approaches look at compositional biases in the complete viral genome or proteome. Compositional representations are also usually drawn from a short list of possibilities such as dinucleotide composition, or a measure of codon bias such as Relative Synonymous Codon Usage (RSCU). The motivation for these representations comes from observed compositional biases across a variety of viral families (Gaunt and Digard, 2022). Especially in the case of codon composition, many computational and experimental results point to the importance of host-virus agreement in the translational environment. For example, virus codon compositions are predictive of their tissue tropism (Hernandez-Alias et al., 2021), and strategies have been discovered in which both the host and viruses manipulate cellular tRNA abundances to either restrict or promote virus replication (Hernandez-Alias et al., 2021; Valdez et al., 2019; Jitobaom et al., 2023; Guo et al., 2021; Jungfleisch et al., 2022). However, this phenomenon has not yet been leveraged for a pathogen-class predictive model to our knowledge. Here, we present our results applying codon frequencies as the feature space for a model capable of distinguishing human-pathogen Orthocoronavirinae examples from non-human-pathogens.

## Results

**FIGURE 1:**
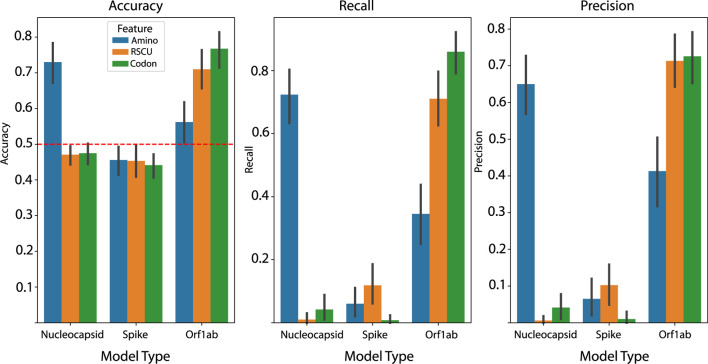
Average performance metrics across each of the one hundred test set splits for each combination of viral CDS and feature representation. Codon frequency model is top performer, with boosts in average performance across each metric over RSCU. Error bars represent 95% confidence interval.

We report that the highest performing models were fit on Orf1ab with a simple codon frequency feature representation, with an average accuracy, recall and precision of 76.74%, 85.96%, and 72.58%, respectively, on sequences from withheld species groups (Figure 1). This was followed closely by the nucleocapsid model using an amino acid frequency representation with 72.99% accuracy and an Orf1ab model using RSCU features with 70.98% accuracy. Our results point to two interesting findings — 1) using only codon frequency as the feature representation results in a modest boost in performance over RSCU (p = 0.024, Wilcoxon rank test) in average accuracy, and 2) depending on the feature representation, information content about host-pathogen potential can vary across the genome with spike protein yielding no models above no-learner in any of the models. As far as we know, these are the only modeling results reported that have relied solely on the Orf1ab or nucleocapsid coding regions.

**FIGURE 2:**
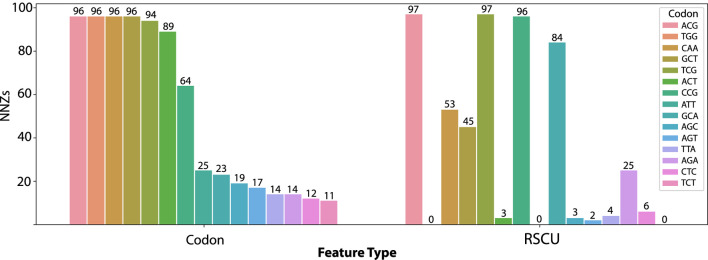
Number of Non-Zero (NNZ) coefficients for the top 15 codons in the codon frequency model across all one hundred models fit on Orf1ab for codon frequency and RSCU features. The Trp^TGG^ codon is used in 96 of 100 codon frequency models but is not available as a feature in the RSCU models.

Since L1-regularization provides feature selection by setting the coefficient of less informative predictors to zero, depending on the strength of regularization term, each model will potentially only use a subset of the total input features. This allows for interpretation of the model results. By counting the number of times predictor had a non-zero coefficient, we can compare which features were utilized most often by the models. First, we observed that 5 codons have non-zero coefficients in almost every codon model fit (>94 of 100), no matter what test set group was withheld. These are Thr^ACG^, Ser^TCG^, Trp^TGG^, Gln^CAA^, and Ala^GCT^ (Figure 2). While there is some overlap in the codons frequently used in the codon frequency representation and RSCU, such as Thr^ACG^ and Ser^TCG^, there are several pronounced differences.

The appearance of the tryptophan codon as a top predictor in the codon frequency model (*which is not a feature of RSCU models as there is only a single codon for tryptophan*) indicates a source of the performance gain. In contrast, while tryptophan amino acid frequency is available to the amino acid models, these models did not perform well on withheld data (56.2% accuracy). This performance advantage is only realized when modeling the interaction between codon frequency features. To illustrate the importance of the tryptophan codon feature, Table 1 shows the non-zero-coefficient predictors in the codon frequency and RSCU models. Several predictors overlap between the two, with the strongest predictor in the codon frequency model being Trp^TGG^.

**TABLE 1:** Predictors and coefficients for model fit without SARS and Alphacoronavirus SC-2013 in both the codon frequency and RSCU representation.

Codon frequency		RSCU	
ACG	−1.0915	TCG	−0.8045
TCG	−0.3325	ACG	−0.4013
GCA	−0.2828	GCA	−0.2395
CTT	−0.0639	CAA	0.1214
AGG	−0.0339		
TTG	0.0809		
AGT	0.1138		
GCT	0.3324		
ACT	0.8509		
CAA	0.9689		
TGG	1.2288		

Another interesting feature of the top three performing models is that they struggle with different subsets of the data. The nucleocapsid model using amino acid frequency features has its performance limited mostly by human coronavirus 229E, where it never successfully recalled the positive class when this virus family was in the test set (N = 12). Meanwhile, the improvement of the codon frequency representation over RSCU in the Orf1ab model is driven primarily by its recall advantage for human coronavirus OC43, where it achieves higher recall in all 22 test splits that contain OC43 as the positive class.

## Methods

### Data collection

Sequence data for this study was gathered from the NCBI Virus database for sequences submitted before September 2018. For SARS-CoV-2, sequences were downloaded and subsampled to include 10 records from each variant of concern (VOCs) as of August 2021: Alpha, Beta, Gamma, and Delta. Viral coding sequences (CDS) were categorized by gene name. For genomes where a full length sequence was available, but no Orf1ab annotation was present, annotations were applied using VAPiD v1.6.7 (Shean et al., 2019) resulting in 215 additional Orf1ab sequences. The sequences that this method was applied to are specified in Supplementary Table S2. This resulted in final datasets for Orf1ab (N = 2,270), Spike (N = 2,198), and Nucleocapsid (N = 2,201). These sequences are available in Supplementary Material (https://doi.org/10.5281/zenodo.14851561).

### Pathogen classification

Sequences were labeled as human-pathogenic if they were associated with known disease in humans, and this label was the “positive class” with respect to the models. This classification included seven positive human-pathogen groups: Human CoV 229E, Human CoV HKU1, MERS-CoV, Human CoV NL63, Human CoV OC43, SARS-CoV-1, and SARS-CoV-2. Non-human-pathogenic representatives included various Alphacoronaviruses, Betacoronaviruses, Gammacoronaviruses, and Deltacoronaviruses. These sequences were labeled as non-human-pathogens unless they caused disease in humans. For example, SARS-CoV-1 isolates from civets and MERS isolates from camels were classified as human pathogens, while all other isolates remained non-human-pathogens (negative class label with respect to the models).

### Cross-validation and test set design

The primary goal was to evaluate the model’s ability to generalize to novel species within the Orthocoronavirinae clade. To achieve this, a group-based, nested-cross-validation strategy was employed. Each sequence was assigned a group label based on its species-level taxonomy ID and pathogen class (human or non-human). For instance, Human CoV OC43 was labeled as “1_694003”(human pathogen representative of Betacoronavirus 1), while Bovine CoV and Porcine Hemagglutinating Encephalomyelitis Virus (PHEV) were labeled as “0_694003” (non-human-pathogen representatives of Betacoronavirus 1). One hundred test-group-splits were performed where each split would include one group randomly selected from each pathogenicity class. All models for each combination of viral CDS and feature type were fit using the same test splits. The groups used for each split are included in Supplementary Table S1.

### Data balancing and supersampling

For each test set split, the remaining sequences were divided into training and validation sets. The sequence data used for modeling had a modest class imbalance of approximately 43% positive class membership. However, due to large imbalances in species representation, such as MERS which represented 435 of the 973 Orf1ab positive class members, some test splits could modulate this imbalance to as low as 29%. Therefore, training data were supersampled to ensure balance between human and non-human-pathogen classes. A two-tiered randomization process first determined whether to sample from the positive or negative class, then uniformly sampled sequences from the corresponding groups. Therefore, each training and test split should be roughly class balanced at 50/50 within each pathogen class and the representatives from each species group uniformly distributed. Training data were super sampled to 4,000 records, while test sets were resampled to 200 records.

### Feature representations

Three feature extraction methods were tested

1. *Amino acid frequency*: Counts of each amino acid in the CDS.
2. *Relative Synonymous Codon Usage (RSCU)*: Codon counts normalized by the number of synonymous codons for each amino acid.
3. *Codon frequency*: Raw counts of each codon, independent of synonymous grouping.

### Model training and selection

All models were trained using L1-regularized logistic regression. To prevent data leakage, preprocessing steps (e.g., standard scaling) were applied within a scikit-learn pipeline (Pedregosa et al., 2011). Cross-validation was performed using a leave-two-groups-out strategy, where one positive and one negative group were withheld for validation. Hyperparameters, including the regularization term and model tolerance, were optimized using randomized cross-validation with 1,000 parameter combinations sampled.

### Model evaluation

Model performance was assessed for the training and test splits using metrics such as accuracy, precision, recall. Model selection in cross validation was guided by negative Brier score. We averaged the performance statistics across each of the one hundred test and training set splits for each combination of viral CDS and feature representation. Persistent model objects for the selected model, a cross-validation report, a list of training set and test set misclassified records, a summary of model parameters, and a table of the non-zero coefficient predictor variables and their coefficients were saved with each model fit for each test set split and are available in Supplementary Material (https://doi.org/10.5281/zenodo.14851561). Code used to produce the models is available in the github repository (https://github.com/mriglobal/codon_amino_cov/tree/main).

## Discussion

These results provide new insights into assumptions underlying viral genotype-to-phenotype modeling. While the specific biological phenomena driving the performance of codon-based models and in the specific context of Orf1ab remain uncertain, the interpretation of these models offers potentially valuable context. In logistic regression models, the coefficients for each predictor reflect the increase or decrease in the log likelihood of the positive class for each unit of the predictor variable. Predictors with positive coefficients are enriched in the positive class (in this case, human pathogens), whereas those with negative coefficients are depleted. Notably, Thr^ACG^ and Ser^TCG^ consistently exhibited negative coefficients across both the codon frequency and RSCU models. These codons are the rarest for their respective amino acids and are among the rarest codons in the human genome, which may be related to their significance in the models. The role of translation-controls in virus-host interactions has been a growing target of research because of virus reliance on host translational machinery to make their proteins (Hoang et al., 2021). We hypothesize that the results presented here are a signature of these translation controls that are host specific. Numerous theoretical factors have been identified to contribute to codon usage bias in viruses, including CpG avoidance for Zinc finger antiviral protein binding (Meagher et al., 2019), RNA secondary structure requirements (Belalov and Lukashev, 2013), and alignment with host translational preferences (Jitobaom et al., 2020). While these models cannot directly elucidate underlying mechanisms, they provide hypotheses for the observed patterns. Relative synonymous codon usage (RSCU) is commonly employed to model translation-influenced systems, such as gene expression (Bahiri-Elitzur and Tuller, 2021). However, in a tRNA supply-and-demand framework, RSCU is insensitive to codon compositional features, such as tryptophan codon frequency, that may influence translation efficiency. Evidence suggests that viruses can manipulate host tRNA pools to enhance replication. For example, Flaviviruses counteract Schlafen-family viral restriction genes (Valdez et al., 2019), HIV alters tRNA abundances to improve translation efficiency (van Weringh et al., 2011), and Influenza A and Vaccinia viruses modulate translationally active tRNA pools at the polysome (Pavon-Eternod et al., 2013). Whether Schlafen-family genes are activated during coronavirus infections or whether TRMT-1 cleavage by the main protease (Zhang et al., 2024) affects cellular tRNA abundances remains unknown. However, data indicate that both tRNA levels and 5-methoxycarbonylmethyl-2-thiouridine modifications of certain position-34-U tRNA isoacceptors are enriched in SARS-CoV-2-infected cells (Eldin et al., 2024), including the Gln^CAA^ codon identified in our models as a significant predictor.

Compositional representations of viral sequences likely reflect aggregated effects from these biological factors. However, assuming uniform selective pressures of these potential sources across viral genomes might be too limiting when modeling their contribution to a phenotype. For example, Influenza A PB1 might have signatures of host codon adaptation that are otherwise absent from the remaining virus segments. A codon adaptation index study on the PB1 gene for seasonal H3N2 Influenza found that the coding region adapted over time to tRNA availabilities in interferon-treated cells (Smith et al., 2018). Coronaviruses are well known for their highly-conserved genomic organization (Fehr and Perlman, 2015). The reason the Orf1ab codon-based representations perform better than those same features in other coronavirus CDSs could be that Orf1ab is particularly sensitive to host translational disagreement in a way that other coding regions are not because it is the initially translated reading frame upon infection. Given knowledge of eukaryotic translational controls such as No-Go mRNA decay (Veltri et al., 2022), it is not difficult to imagine the consequences of engaging premature mRNA decay for a single-stranded positive sense RNA virus. Additionally, if coronaviruses are modifying the host translational environment to be pro-viral, proteins such as the polyprotein that need to be translated before these modified conditions can be achieved might represent an early selection bottleneck in the infection cycle. While there may be general compositional similarities among human-infecting viruses, these results suggest that predictive power in genotype-to-phenotype models can be achieved through feature engineering. The models presented here demonstrate substantial information content regarding human-pathogenic potential in specific genomic contexts. However, they remain incomplete, as many known barriers to zoonosis in coronaviruses are not addressed. These barriers include, for example, cross-reactivity to endogenous human coronaviruses in the case of AlphaCoV1 (Tortorici et al., 2022) and 229E-related Camel alphacoronaviruses (Corman et al., 2016), as well as host-receptor binding compatibility.

Limitations of the approach described in this study primarily stem from the assumption of the labeling scheme. The results presented are under the assumptions described in Methods about human pathogenicity in the observed sequence data. However, the model labels employed here encompass both the ability to cause disease in humans and consequently be observed. There may be various other categories that are not modeled by the response variable here including the ability to infect and not cause disease, or spill over and cause disease but not result in forward transmission (and therefore not observed). Additionally, as previously mentioned, we sought to identify a model that applied across all of the Orthocoronvirinae family. One advantage the codon frequency approach may benefit from in this situation is that all of the known human coronaviruses are respiratory viruses. Depending on the virus family, this approach might fail, given the identified connection between codon composition and tissue tropism (Hernandez-Alias et al., 2021). Thus, an expectation of similarities in codon frequencies to be observable in the genes of, for instance, all Flaviviruses that infect humans, might not be valid, since virus species in this family can infect many different human tissues. Nevertheless, the findings here suggest that ensemble models incorporating these approaches could improve predictive power in coronaviruses. More broadly, general genotype-to-phenotype modeling efforts in viruses could benefit from similar strategies.

## Data Availability

The original contributions presented in the study are included in the article/Supplementary Material, further inquiries can be directed to the corresponding author.
