# Supplementary material for: ORF1ab codon frequency model predicts host-pathogen relationship in orthocoronavirinae
Source: Front Bioinform. 2025 Mar 18;5:1562668. doi: 10.3389/fbinf.2025.1562668 (PMC11958986; doi:10.3389/fbinf.2025.1562668)
Supplement: Supplementary file 1 [file Table2.docx]

| Split # | Positive Group | Negative Group |
| --- | --- | --- |
| 1 | 1_11137 | 0_694014 |
| 2 | 1_290028 | 0_1159906 |
| 3 | 1_694003 | 0_694003 |
| 4 | 1_290028 | 0_1159908 |
| 5 | 1_11137 | 0_2501928 |
| 6 | 1_694009 | 0_11137 |
| 7 | 1_694003 | 0_1159906 |
| 8 | 1_694003 | 0_694009 |
| 9 | 1_1335626 | 0_1160968 |
| 10 | 1_2697049 | 0_693998 |
| 11 | 1_290028 | 0_1739625 |
| 12 | 1_290028 | 0_1297661 |
| 13 | 1_290028 | 0_2501926 |
| 14 | 1_694009 | 0_1159903 |
| 15 | 1_694003 | 0_2032731 |
| 16 | 1_290028 | 0_694008 |
| 17 | 1_11137 | 0_1297661 |
| 18 | 1_11137 | 0_574549 |
| 19 | 1_11137 | 0_694006 |
| 20 | 1_1335626 | 0_2501929 |
| 21 | 1_2697049 | 0_1965089 |
| 22 | 1_2697049 | 0_2032731 |
| 23 | 1_277944 | 0_28295 |
| 24 | 1_1335626 | 0_1159904 |
| 25 | 1_694003 | 0_2501929 |
| 26 | 1_11137 | 0_694007 |
| 27 | 1_694009 | 0_11137 |
| 28 | 1_694003 | 0_694000 |
| 29 | 1_694003 | 0_1503293 |
| 30 | 1_694009 | 0_2501928 |
| 31 | 1_694003 | 0_1739625 |
| 32 | 1_694003 | 0_2501929 |
| 33 | 1_694009 | 0_1297661 |
| 34 | 1_277944 | 0_694003 |
| 35 | 1_1335626 | 0_2501961 |
| 36 | 1_694009 | 0_2501928 |
| 37 | 1_1335626 | 0_2501960 |
| 38 | 1_11137 | 0_694005 |
| 39 | 1_694003 | 0_1335626 |
| 40 | 1_290028 | 0_2032731 |
| 41 | 1_11137 | 0_694006 |
| 42 | 1_2697049 | 0_694000 |
| 43 | 1_2697049 | 0_572290 |
| 44 | 1_1335626 | 0_1965089 |
| 45 | 1_1335626 | 0_693999 |
| 46 | 1_694003 | 0_1739625 |
| 47 | 1_694003 | 0_1159902 |
| 48 | 1_2697049 | 0_1159903 |
| 49 | 1_277944 | 0_693997 |
| 50 | 1_1335626 | 0_1508228 |
| 51 | 1_11137 | 0_1297661 |
| 52 | 1_277944 | 0_693997 |
| 53 | 1_694003 | 0_2501928 |
| 54 | 1_1335626 | 0_1244203 |
| 55 | 1_290028 | 0_2501927 |
| 56 | 1_1335626 | 0_694003 |
| 57 | 1_694009 | 0_694009 |
| 58 | 1_290028 | 0_694000 |
| 59 | 1_290028 | 0_1913643 |
| 60 | 1_277944 | 0_1159903 |
| 61 | 1_694003 | 0_1964806 |
| 62 | 1_277944 | 0_1297661 |
| 63 | 1_694009 | 0_1264898 |
| 64 | 1_2697049 | 0_1159907 |
| 65 | 1_694003 | 0_574549 |
| 66 | 1_1335626 | 0_11137 |
| 67 | 1_1335626 | 0_1964806 |
| 68 | 1_1335626 | 0_694007 |
| 69 | 1_694009 | 0_1739625 |
| 70 | 1_694003 | 0_1739625 |
| 71 | 1_694003 | 0_1264898 |
| 72 | 1_694003 | 0_694003 |
| 73 | 1_277944 | 0_28295 |
| 74 | 1_277944 | 0_1159906 |
| 75 | 1_277944 | 0_2032731 |
| 76 | 1_277944 | 0_11137 |
| 77 | 1_694009 | 0_1335626 |
| 78 | 1_1335626 | 0_1159902 |
| 79 | 1_11137 | 0_1965093 |
| 80 | 1_290028 | 0_694003 |
| 81 | 1_2697049 | 0_693998 |
| 82 | 1_11137 | 0_1508224 |
| 83 | 1_1335626 | 0_572290 |
| 84 | 1_290028 | 0_1503293 |
| 85 | 1_2697049 | 0_1503293 |
| 86 | 1_2697049 | 0_1244203 |
| 87 | 1_290028 | 0_1335626 |
| 88 | 1_1335626 | 0_694006 |
| 89 | 1_694003 | 0_2501926 |
| 90 | 1_694009 | 0_1965093 |
| 91 | 1_694003 | 0_1335626 |
| 92 | 1_2697049 | 0_694014 |
| 93 | 1_694009 | 0_1965093 |
| 94 | 1_277944 | 0_1964806 |
| 95 | 1_694003 | 0_1159907 |
| 96 | 1_694003 | 0_2501929 |
| 97 | 1_11137 | 0_1508224 |
| 98 | 1_290028 | 0_1297661 |
| 99 | 1_277944 | 0_11137 |
| 100 | 1_1335626 | 0_1335626 |

Supplementary Table 1. Test Splits used for model evaluation.
